# Supplementary material for: Proportion of School Attending Adolescents Meeting the Recommended Moderate-to-Vigorous Physical Activity Level and Its Predictors in Lagos, Nigeria
Source: Int J Environ Res Public Health. 2021 Oct 13;18(20):10744. doi: 10.3390/ijerph182010744 (PMC8536101; doi:10.3390/ijerph182010744)
Supplement: Supplementary file 1 [file ijerph-18-10744-s001.zip › ijerph-1405584-supplementary.pdf]

International Journal of
*Environmental Research and Public Health*

MDPI

**Table S1.** Comparison of participants with and without missing MVPA level data by predictors.

| | With missing MVPA level data ($n$ = 192) $n$ (%) or otherwise indicated | Without missing MVPA level data ($n$ = 528) $n$ (%) or otherwise indicated | $p$-value |
|---|---|---|---|
| Age (years) | 15.2 (1.5)* | 14.9 (1.6)* | 0.005 |
| Sex | | | 0.271 |
| Male | 96 (50.3) | 234 (44.3) | |
| Female | 95 (49.7) | 292 (55.3) | |
| Prefer not to say | 0 | 2 (0.4) | |
| Ethnicity | | | 0.141 |
| Hausa | 3 (1.6) | 6 (1.1) | |
| Ibo | 48 (25.1) | 172 (32.6) | |
| Yoruba | 110 (57.6) | 291 (55.2) | |
| Others | 30 (15.7) | 58 (11.0) | |
| Socioeconomic status | | | 0.025 |
| Low | 95 (51.1) | 229 (44.3) | |
| Middle | 56 (30.1) | 213 (41.2) | |
| High | 35 (18.8) | 75 (14.5) | |
| School | | | 0.705 |
| Public | 113 (58.9) | 319 (60.4) | |
| Private | 79 (41.2) | 209 (39.6) | |
| Class | | | <0.001 |
| Junior | 44 (22.9) | 231 (43.8) | |
| Senior | 148 (77.1) | 297 (56.3) | |
| BMI | | | 0.569 |
| Grade I-III thinness | 35 (19.3) | 111 (21.1) | |
| Normal | 127 (70.2) | 356 (67.7) | |
| Overweight | 14 (7.7) | 51 (9.7) | |
| Obese | 5 (2.8) | 8 (1.5) | |
| Waist-to-hip ratio | 0.8 (0.05) | 0.8 (0.04) | 0.378 |
| Sedentary behaviour | | | 0.402 |
| Low | 78 (46.2) | 214 (42.5) | |
| High | 91 (53.8) | 290 (57.5) | |
| Self-efficacy | 3.5 (0.6)* | 3.6 (0.7)* | 0.311 |
| Perceived benefits | 3.8 (0.6)* | 3.8 (0.6)* | 0.416 |
| Perceived barriers | 2.7 (0.6)* | 2.6 (0.6)* | 0.413 |

* Mean (SD).
